# Cellular unfolded protein response against viruses used in gene therapy

**DOI:** 10.3389/fmicb.2014.00250

**Published:** 2014-05-26

**Authors:** Dwaipayan Sen, Balaji Balakrishnan, Giridhara R. Jayandharan

**Affiliations:** ^1^Department of Hematology, Christian Medical CollegeVellore, India; ^2^Centre for Stem Cell Research, Christian Medical CollegeVellore, India

**Keywords:** gene therapy, UPR, ER-stress, ER-homeostasis, viral vectors, chaperones

## Abstract

Viruses are excellent vehicles for gene therapy due to their natural ability to infect and deliver the cargo to specific tissues with high efficiency. Although such vectors are usually “gutted” and are replication defective, they are subjected to clearance by the host cells by immune recognition and destruction. Unfolded protein response (UPR) is a naturally evolved cyto-protective signaling pathway which is triggered due to endoplasmic reticulum (ER) stress caused by accumulation of unfolded/misfolded proteins in its lumen. The UPR signaling consists of three signaling pathways, namely PKR-like ER kinase, activating transcription factor 6, and inositol-requiring protein-1. Once activated, UPR triggers the production of ER molecular chaperones and stress response proteins to help reduce the protein load within the ER. This occurs by degradation of the misfolded proteins and ensues in the arrest of protein translation machinery. If the burden of protein load in ER is beyond its processing capacity, UPR can activate pro-apoptotic pathways or autophagy leading to cell death. Viruses are naturally evolved in hijacking the host cellular translation machinery to generate a large amount of proteins. This phenomenon disrupts ER homeostasis and leads to ER stress. Alternatively, in the case of gutted vectors used in gene therapy, the excess load of recombinant vectors administered and encountered by the cell can trigger UPR. Thus, in the context of gene therapy, UPR becomes a major roadblock that can potentially trigger inflammatory responses against the vectors and reduce the efficiency of gene transfer.

## Introduction

One of the important functions of cellular metabolism is protein folding. Endoplasmic reticulum (ER) is the site where all the proteins (secreted, membrane bound, and organelle targeted proteins) are typically processed and folded in eukaryotes (Kaufman et al., 2002; Naidoo, 2009). This accumulates a very high concentration of proteins in the ER which can lead to co-aggregation between proteins and/or polypeptides (Stevens and Argon, 1999). Therefore, the lumen of the ER needs a unique environment that promotes processing of proteins but prevents their aggregation (Anelli and Sitia, 2008; Kim et al., 2008; Hetz et al., 2011; Hetz, 2012). Sometimes, due to a high demand in protein synthesis due to various physiological reasons, the processing capacity of the ER can be challenged (Zhang and Kaufman, 2006; Marcinak and Ron, 2010; Hetz et al., 2011). This results in an imbalance in the ER environment, which is referred to as ER stress (Liu and Howell, 2010; Marcinak and Ron, 2010; Hetz et al., 2011; Iwata and Koizumi, 2012). Altered protein folding leading to ER stress can be induced by various factors such as glucose deprivation, aberrant calcium regulation, viral infection and hypoxia. Normally, cells ensure that proteins are correctly folded using a combination of molecular chaperones namely, the foldases and lectins (Naidoo, 2009). If unfolded or misfolded proteins continue to accumulate, eukaryotes induce the UPR. The basic goal of UPR is to recover the (lost) homeostasis (adaptation), reduce stress within the ER compartment and prevent any cytotoxic effect that might be caused by misfolded proteins *via* adaptive mechanisms as well as by blocking mRNA translation (Xu et al., 2005; Kim et al., 2008; Ye et al., 2011). During adaptation, the UPR tries to correct folding homeostasis *via* induction of chaperones that promote protein folding (Meusser et al., 2005; Kim et al., 2008). However, when proper folding cannot be restored, incorrectly folded proteins are targeted to ER Associated Degradation (ERAD) pathways for processing (Kaufman et al., 2002). UPR is also known to trigger several molecules of the innate immunity pathway, most notably mitogen- activated protein kinases, p38 and nuclear factor-κ B (NF-κ B) which collectively trigger the UPR induced alarm signal (Ron and Walter, 2007; Kim et al., 2008; Tabas and Ron, 2011) to remove translational block and down-regulate the expression and activity of pro-survival factors such as the B-cell lymphoma 2 (Bcl2) protein. However, if the function of the ER cannot be re-established, UPR eliminates the damaged cells by apoptosis or autophagy (Bernales et al., 2006; Kamimoto et al., 2006; Yorimitsu et al., 2006; Hoyer-Hansen et al., 2007; Kouroku et al., 2007). Apart from such a response against *de novo* synthesized proteins in a cell, the massive accumulation of exogenous proteins intra-cellularly as in the case of viral infection is also known to contribute to ER stress responsive pathways (Zhang and Wang, 2012).

For a virus to successfully infect mammalian cells, it has to undergo several aspects in its life-cycle-their attachment to cell surface receptors, endocytosis, intracellular trafficking, polypeptide synthesis and genome replication (Balakrishnan and Jayandharan, 2014). Viruses are naturally evolved to utilize host cell machinery to successfully complete their life cycle and during this process they produce several viral proteins within host cells. As a natural response to these foreign proteins, the cell in turn can activate the UPR and interferon response. Thus, a potential mechanism that can limit viral replication is the UPR. It is not surprising that viruses have also evolved mechanisms to manipulate UPR pathways to facilitate their infection (Zhang and Wang, 2012). This generally involves regulation of stress response proteins and several molecular chaperones to modulate UPR and increase ER folding capacity or by induction of translational attenuation to repress the UPR pathways (Zhang and Wang, 2012). Several viruses like adenovirus (Ad), adeno-associated virus (AAV), dengue virus, cytomegalovirus, respiratory syncytial virus, simian virus-5, Tula virus, rota virus African swine fever virus, herpes simplex virus type 1 (HSV-1), hepatitis C virus, corona virus, influenza virus amongst others have been shown to regulate host cell UPR machinery to promote their infection and persistence in the host (Bitko and Barik, 2001; Netherton et al., 2004; Isler et al., 2005; Paradkar et al., 2011; Pena and Harris, 2011; Zhang and Wang, 2012). For example, rotavirus interrupts the inositol requiring protein-1 (IRE1) and activating transcription factor 6 (ATF6) UPR pathways by translational inhibition through its non-structural protein NSP3 (Trujillo-Alonso et al., 2011). Hepatitis C virus (HCV) has been shown to suppress the IRE1-XBP1 pathway to promote its expression and persistence in the liver (Tardif et al., 2004). Likewise cytomegalovirus uses the viral protein M50 to downregulate IRE1 leading to suppression of UPR (Stahl et al., 2013). This article reviews the tug of war that is initiated by the cell through its UPR signaling against viruses used in gene therapy and dissects how this information can be helpful to improve gene delivery strategies.

## UPR pathways

**Figure 1:**
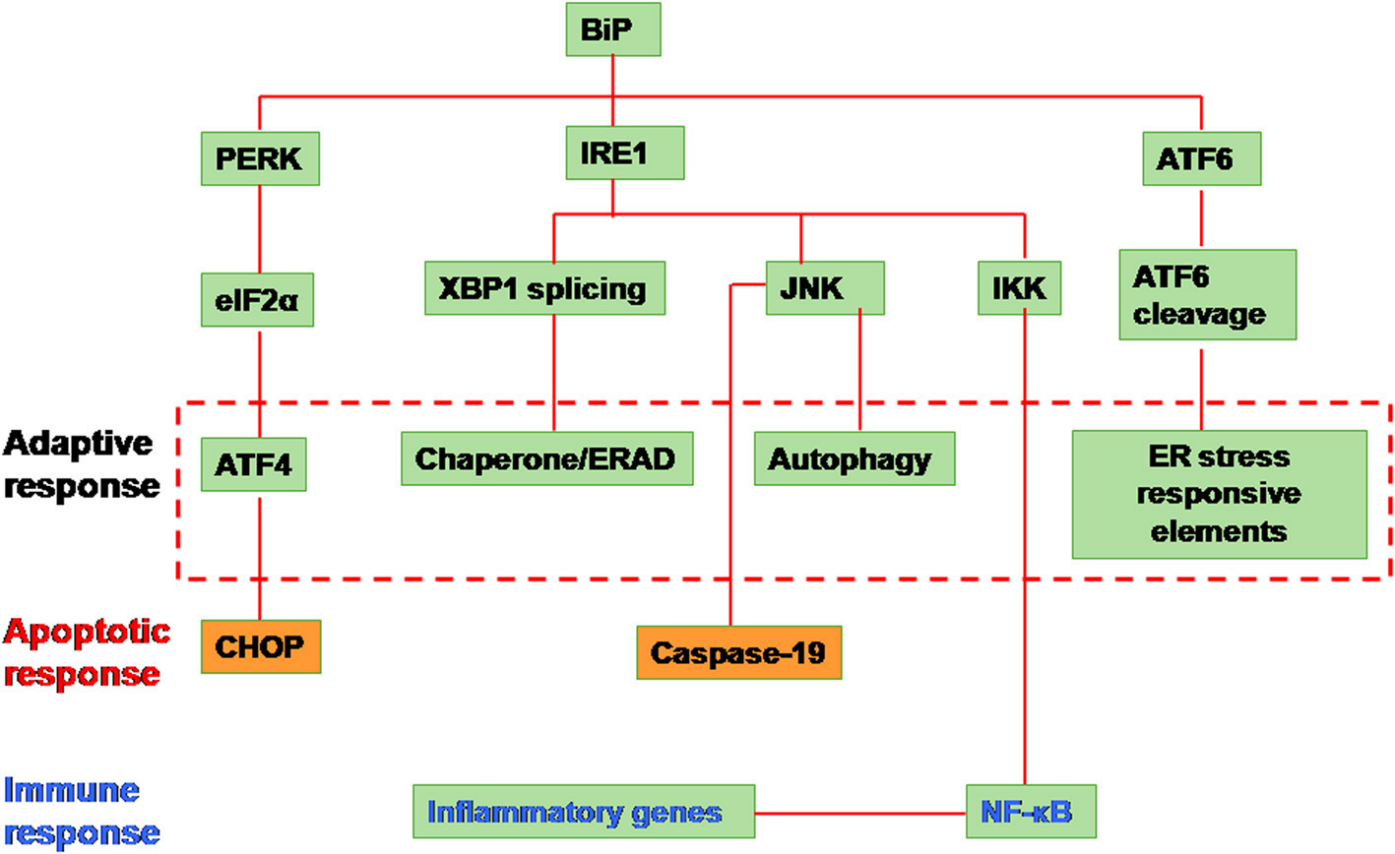
**Unfolded protein response signaling**. The signaling is initiated by the activation of the proximal sensors of the unfolded protein response (UPR) namely, (1) protein kinase R (PKR)-like ER kinase (PERK), (2) activating transcription factor (ATF) 6 and (3) inositol-requiring enzyme 1 (IRE1). A protein called immunoglobulin heavy chain binding protein (BiP) functions as the master regulator. BiP under normal conditions remains attached to all the three sensors in the luminal domain of the endoplasmic reticulum (ER). Upon encountering any stress like accumulation of misfolded/unfolded proteins or a massive inflow of any exogenous proteins into the ER, the stress sensors, PERK, IRE1, and ATF6, are activated by the release of BiP from the sensors leading to any of the three distinct pathways. (1) When PERK is activated, it dimerises and autophosphorylates leading to phosphorylation of the eukaryotic translation initiation factor (elF) 2α. Activated elF2α represses global protein translation of the cell. However the downstream protein called ATF 4 can escape translational repression since it has upstream open reading frames leading to its activation. The activated ATF4 translocates into the nucleus activating a set of target genes to restore cellular homeostasis (adaptive response). However in situations when the cellular homeostasis cannot be restored, C/EBP homologous protein (CHOP) is activated leading to apoptosis. (2) When IRE1 is activated, it dimerizes and autophosphorylates leading to the activation of its endoribonuclease activity. This leads to an unusual splicing of XBP1 (X-box binding protein 1) cleaving 26 nucleotide intron within. The Spliced XBP1 (sXBP1) protein translocates to nucleus transcribing chaperones and unfolded protein response elements (UPREs) to restore cellular homeostasis. In some cases, the IRE1 activates the cellular JNK through phosphorylation. This activated JNK either leads to apoptosis by activaton of caspase 19 or leads to autophagy. Alternatively, IRE1 activates IKK by interacting with tumor necrosis factor receptor-associated factor 2 (TRAF2) which phosphorylates Iκ B. This releases nuclear factor (NF)-κ B. The activated NF-κ B translocates into the nucleus and transcribes inflammatory genes. (3) Activation of the third sensor of UPR, ATF6 leads to its translocation into the Golgi complex. In the golgi complex, ATF6 will be cleaved by proteases such as site-1 protease (S1P) and S2P. This cleaved ATF6 fragment further transcribes chaperones and UPRE to cope with the cellular stress and restore homeostasis (Yoshida et al., 2001; Lee et al., 2002; Harding et al., 2003; Novoa et al., 2003; Wu et al., 2007; Yamamoto et al., 2007; Raven et al., 2008).

Three branches of the UPR have been characterized, which are mediated by ER-located transmembrane proteins: IRE1, protein kinase RNA-like ER kinase (PERK) and ATF6. The binding immunoglobulin protein (BiP) is the master regulator of the UPR. All the three arms of UPR are held in an inactive state by the binding of the BiP to their N-terminal region of IRE1, PERK and ATF6 proteins. When the cell encounters stress, BiP is released due to competitive binding of the misfolded proteins and thus leading to activation of UPR signaling (Figure 1) (Xu et al., 2005).

### IRE1 pathway

IRE1, the most evolutionarily conserved branch of UPR (Cox et al., 1993) initiates both the pro-survival and pro-apoptotic components in the presence of misfolded proteins. In mammals two isoforms of IRE1 have been identified, IRE1α and IRE1β ; IRE1α is expressed in a variety of tissues (Tirasophon et al., 1998), whereas IRE1β is primarily found in the intestine and lung (Bertolotti et al., 2001; Martino et al., 2013). Mechanistically, when there is an increase in unfolded or misfolded protein load, the BiP molecule interacts with the N-terminus of IRE1, located in the ER lumen. This sensing leads to dimerization of IRE-1 and activates two distinct signaling arms of the IRE-1 pathway. The early signaling occurs through the cleavage of a 26-nucleotide intron from the XBP1-mRNA (Shen et al., 2001; Yoshida et al., 2001; Lee et al., 2002; Malhotra and Kaufman, 2007) generating a 41 kDa frameshift variant (sXBP1). sXBP1 acts as a potent transcription factor that regulates the expression of several protein degradation related genes (Rao and Bredesen, 2004; Malhotra and Kaufman, 2007). The late signaling sensor of IRE1 is initiated when the cytosolic IRE1α dimers interact with molecules like the tumor necrosis factor receptor-associated factor 2 (TRAF2) which activates the signal-regulating kinase (ASK1) and further activation of cJUN NH2-terminal kinase (JNK) and p38MAPK (Urano et al., 2000). These proteins in turn trigger a proapoptotic signal through pro-apoptotic molecules such as Bim and caspase-3 leading to cell death.

### PERK pathway

PERK is an ER-localized type I transmembrane protein containing a catalytic kinase domain homologous to other kinases of the eukaryotic translation initiation factor 2 (eIF2) such as general control non-depressible-2 (GCN2), heme-regulated inhibitor (HRI) and protein kinase R (PKR) (Harding et al., 1999). The luminal stress sensor domain of PERK is structurally and functionally homologous with the luminal domain of IRE1α, implicating very similar stress-sensing mechanisms between PERK and IRE1α (Bertolotti et al., 2000). The PERK branch of UPR transduces both the pro-survival as well as pro-apoptotic signals following the accumulation of unfolded or misfolded proteins in the ER. However, its main function is to modulate translation. During initial stages of ER stress, PERK oligomerizes in the ER membrane and induces autophosphorylation (He, 2006). Activated PERK phosphorylates eIF2α at S51 (Harding et al., 1999; Raven et al., 2008) leading to global attenuation of translational machinery, thus reducing the trafficking of newly synthesized proteins into the already stressed ER compartment. The accumulated protein load is then cleared off from the ER by ERAD pathway with simultaneous expression of pro-survival genes like activating transcription factor 4 (ATF4) (Harding et al., 2003). ATF4 is not affected by the global eIF2α translational block because of the presence of internal ribosome entry site (IRES) sequences in the 5′ untranslated regions (Schroder and Kaufman, 2005). However ATF4 can drive the cell toward apoptosis by inducing expression of factors like C/EBP homologous protein (CHOP) and growth arrest and DNA damage-inducible protein 34 (GADD34) (Zinszner et al., 1998; Novoa et al., 2003).

### ATF6 pathway

ATF6 is a type II ER transmembrane protein belonging to the bZIP family of transcription factors. The ER luminal domain acts as the sensor for ER stress due to the protein overload while the cytoplasmic domain acts as a transcription factor (49). ATF6 has two homologs- ATF6α (Hai et al., 1989; Haze et al., 1999) and ATF6β (Min et al., 1995; Khanna and Campbell, 1996; Haze et al., 2001) with redundant roles in UPR. Upon dissociation of BiP from the N-terminus of ATF6 following ER stress, it translocates from the ER to the golgi where it is cleaved by resident proteases like site 1 protease (S1P) and site protease (S2P) (Hetz et al., 2011) to release its cytoplasmic DNA binding fragment called ATF6f. ATF6f increases degradation of unfolded proteins as well as induces the activity of several ER chaperone proteins like BiP, protein disulfide isomerase (PDI) and ER degradation-enhancing alpha-mannosidase-like protein 1 (EDEM1) (Wu et al., 2007; Yamamoto et al., 2007).

## Gene therapy

In the last two decades, gene therapy has been immensely popular to treat various inherited as well as acquired disorders (Kay, 2011; Misra, 2013). Gene therapy involves either replacing a mutated gene with a healthy copy or introducing a new gene into the cells to help protect against the disease. Despite significant success seen in the treatment of diseases such as lipoprotein lipase deficiency (Gaudet et al., 2012), haemophilia B (Manno et al., 2006; Nathwani et al., 2011), Leber's Congenital Amaurosis (Simonelli et al., 2010) or severe combined immunodeficiency (SCID) (Cavazzana-Calvo et al., 2000), the safety and efficacy of this novel modality of treatment recognizably needs to be improved. For a clinically relevant gene therapy protocol, the efficient delivery and optimal expression of the gene of interest are very important. Since viruses are naturally evolved to efficiently infect and transfer DNA into the host, engineered (gutted) viruses are the most desirable as gene delivery vehicles. Viral vectors currently available for gene therapy can roughly be categorized into integrating and non-integrating vectors. Vectors based on retroviruses (including lentivirus and foamy virus) have the ability to integrate their viral genome into the chromosomal DNA of the host cell, which can theoretically achieve life-long gene expression. Vectors based on Ad, AAV and HSV-1 represent the non-integrating vectors (Table 1). These vectors deliver their genomes into the nucleus of the target cell, where they continue to remain episomal. Viral vectors derived from retroviruses, Ad, AAV and HSV have been employed in the majority of gene therapy clinical trials (Table 2). Recognizing the activation and basis of cellular events like UPR in response to a virus used in gene therapy is important to further optimize gene delivery.

**Table 1:** **Characteristics of the common viruses used in gene therapy**.

**Viral Vector**	**Description**	**Associated disease**	**Maximum transgene capacity**	**Host genome integration**	**Transduction of cells**	**Advantages**	**Disadvantages**
Adenovirus	36 kb dsDNA, non-enveloped, icosahedric, 70–90 nm in diameter	Yes	~30 kb	No	Both dividing and non-dividing	Easy production of high titres, ability to infect a wide range of cell types, capacity to carry large transgene	Adverse host humoral and cellular immune response, transient gene expression
Retroviruses (retrovirus and lentivirus)	7–10 kb ssRNA, enveloped, ~100 nm diameter	Yes	~8 kb	Yes	Both dividing and non-dividing	High infection efficiency, stable and permanent gene transfer	Insertional mutagenesis causing cancer, high immunogenicity
Adeno associated virus (AAV)	4.7 kb ssDNA, Icosahedric, non-enveloped, ~22 nm diameter	No	~4.7 kb	No	Both dividing and non-dividing	Low immunogenicity, non-infectious	Limited transgene carrying capacity, not suitable to target rapidly dividing cells
Herpesvirus-HSV-1	~152 kb dsDNA, icosahedric enveloped, ~125 nm diameter	Yes	~150 kb	No	Only dividing cells	Large transgene carrying capacity, production of high titres	Host immune response, short term gene expression

**Table 2:** **Viral vectors used in clinical trials (Last date of access—24th March, 2014)**.

**Viral vectors**	**Disease target**	**Clinicaltrials.gov identifier**	**Last update**
**Adenovirus**	Cystic fibrosis	NCT00004779	June 23, 2005
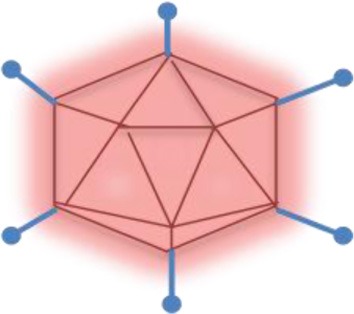	Ovarian cancer	NCT00964756; NCT00562003	February 11, 2013; January 25, 2011
	Metastatic breast cancer	NCT00307229; NCT00197522	May 31, 2012; October 31, 2012
	Lung cancer	NCT00776295	January 16, 2013
	Brain tumor	NCT00004080	February 6, 2009
	melanoma	NCT01397708	March 11, 2014
	Bladder cancer	NCT00003167	January 22, 2013
**Adeno-associated virus (AAV)**	Retinal disease	NCT01482195	November 29, 2011
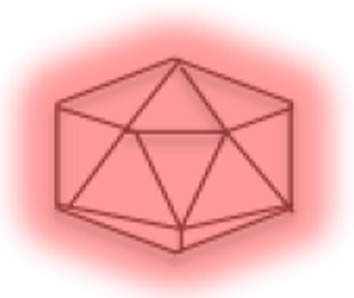	Pompe disease	NCT00976352	December 13, 2013
	Late infantile neuronal ceroid lipofuscinosis	NCT01161576	November 5, 2013
	Leber congenital amaurosis	NCT00749957; NCT00643747; NCT00999609; NCT00516477	March 6, 2013; December 13, 2013; January 13, 2014; January 13, 2014
	Alpha-1 antitrypsin deficiency	NCT01054339; NCT00377416; NCT00430768	March 6, 2013; December 20, 2013; December 20, 2013
	Cystic fibrosis	NCT00004533	June 23, 2005
	Idiopathic Parkinson's disease	NCT00985517	December 10, 2012
	Hemophilia B	NCT01687608; NCT01620801; NCT00076557; NCT00515710; NCT00979238	September 19, 2013; December 20, 2013; April 2, 2007; December 20, 2013; December 20, 2013
	Duchenne muscular dystrophy	NCT00428935	February 4, 2013
	Lipoprotein lipase deficiency	NCT01109498; NCT00891306	September 29, 2011; September 28, 2011
**Herpes simplex virus vectors**	Melanoma, liver cancer, pancreatic cancer, lung cancer	NCT01935453	August 30, 2013
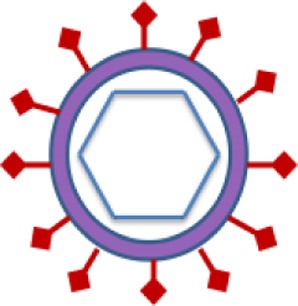	Refractory non-central nervous system (non-CNS) solid tumors	NCT00931931	November 5, 2013
	Head and neck cancer or solid tumors	NCT01017185	February 18, 2013
**Lentivirus**	Lymphoma	NCT00569985	January 6, 2014
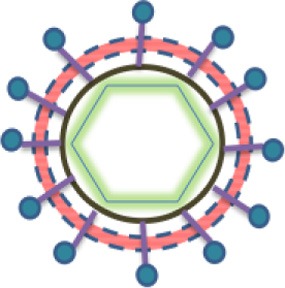	Acute myeloid leukaemia	NCT00718250	July 16, 2008
	ADA-deficient severe combined immunodeficiency (ADA-SCID)	NCT01852071	January 14, 2014
	X-linked severe combined immunodeficiency (X-SCID)	NCT01306019	March 14, 2014
	Fanconi anaemia	NCT01331018	March 6, 2014
	Wiskott—aldrich syndrome	NCT01515462	January 18, 2012
	AIDS-related non-hodgkin lymphoma	NCT01961063	October 30, 2013
**Retrovirus**	Chronic granulomatous disease	NCT00778882	January 15, 2014
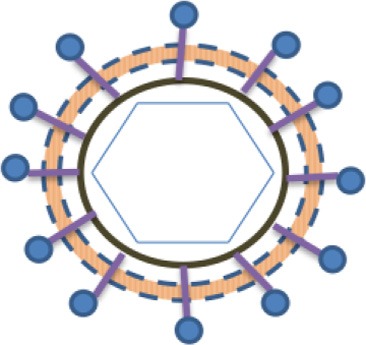	CNS tumors	NCT00005796	October 22, 2009
	X-Linked severe combined immunodeficiency (X-SCID)	NCT00028236	July 26, 2011
	ADA-deficient severe combined immunodeficiency (ADA-SCID)	NCT00598481; NCT00599781; NCT00794508	December 12, 2013; January 23, 2008; February 7, 2013
	Leukocyte adherence deficiency	NCT00023010	December 14, 2010
	Chronic granulomatous disease (CGD)	NCT00564759; NCT00001476	November 27, 2007; December 14, 2010
	Gaucher's disease	NCT00001234	March 3, 2008
	Sickle cell anaemia and β-thalassemia	NCT00669305	December 20, 2013
	Mild hunter synrome	NCT00004454	June 23, 2005
	HIV infection	NCT00001535	February 7, 2008
	Gyrate atrophy	NCT00001735	March 3, 2008

### HSV and UPR

HSV-1 is a large (~152 kb) fast replicating, enveloped, double stranded (ds) DNA virus. The mature viral particle consists of 3 components- an external envelope made of about 13 glycoproteins which helps the virus to bind and enter the host cell; a second layer called tegument which contains 20 different structural and regulatory proteins and finally an icosohedral capsid containing the genetic material. HSV is an attractive choice as a gene therapy vector for various reasons, including its broad tropism, host range and its cellular receptors (Arii et al., 2009; Fan et al., 2009; Wang et al., 2009b), their ability to infect non-dividing cells with high efficiency, high production titers for recombinant particles and a stable/long-term expression of therapeutic genes especially in neurons (Norgren and Lehman, 1998). Three types of HSV-1 vectors are currently in use in gene therapy- replication-defective, replication-competent vectors and amplicons. Deleting one or more genes involved in the lytic cycle creates a replication-defective vector. Replication-competent viruses are attenuated for genes that are not essential for replication *in vitro* (Hu and Coffin, 2003; Post et al., 2004). The amplicons are derived from engineered plasmids, which contain both the HSV packaging recognition sequence (pac) and the origin of replication (ori). These amplicons can be efficiently packaged in mammalian cells as concatamers with the help of HSV helper elements. Also, amplicons are non-toxic and can carry very large DNA fragments of upto 152 kb (Epstein, 2009). Both replication defective and replication competent HSV vectors have been used in gene therapy of several neurological disorders (Table 1). Replication defective HSV vectors have been shown to efficiently transduce both dividing and non-dividing cells including tumors. Taking advantage of this property, HSV vector have been engineered to deliver anticancer transgenes into tumour cells such as melanoma (Krisky et al., 1998; Niranjan et al., 2003), gliosarcoma (Moriuchi et al., 2002; Niranjan et al., 2003) or glioblastoma (Niranjan et al., 2000).

One of the major factor that negatively affects HSV mediated gene delivery is the host immune response directed against it, including the innate and adaptive responses (Ryan and Federoff, 2009). As a first line of defense, innate immunity is a major rate-limiting factor in HSV transduction. One of the principal effector underlying anti-HSV innate defense, is the process of autophagy that is initiated through the cellular UPR pathway (Lee et al., 2009).

**Figure 2:**
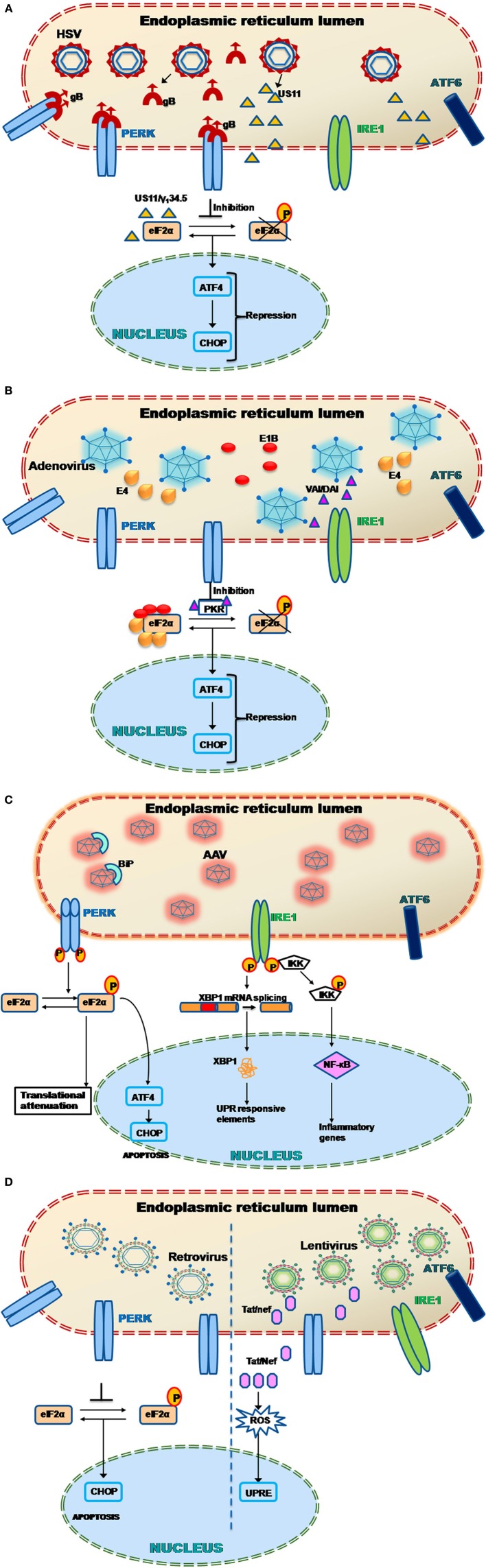
**(A)** Herpes simplex virus (HSV-1) and UPR. HSV-1 produces proteins such as glycoprotein B (gB) and US11 that have been shown to evade the host UPR mechanism (Mulvey et al., 2003, 2007). In particular, the protein gB specifically binds to the PERK proteins preventing their phosphorylation. This leads to PERK inactivation and hence the downstream effector protein elF2α could not get activated leading to ATF4 repression. Alternatively another viral protein called US11 represses the elF2α phosphorylation by directly binding to it. The late HSV viral protein γ_1_34.5 also induces dephosphorylation of eIF2α with the help of the cellular phosphatase PP1α (He et al., 1996). This leads to early repression of ATF4 and CHOP genes downstream. Thus the host UPR response is attenuated and leads to successful viral transduction. **(B)** Adenovirus (Ad) and UPR. Adenoviruses during their late phase of their infection, try to overcome the cellular stress response by preventing the shutdown of protein translation through PKR mediated inhibition of elF2α phosphorylation *via* viral associated RNA molecule I (VAI RNA) as well as double-stranded RNA-activated inhibitor (DAI) (Huang and Schneider, 1990; Mathews and Shenk, 1991; McKenna et al., 2006). Other Ad proteins such as E1B and E4 has also been found to directly bind to the elF2α, thus preventing its phosphorylation and activation of downstream UPR related genes like ATF4 and CHOP (Spurgeon and Ornelles, 2009). **(C)** Adeno associated virus (AAV) and UPR. When the cellular ER encounters AAV particles, specific stress sensors, PERK and IRE1 gets activated (Balakrishnan et al., 2013). PERK phosphorylation leads to the activation of the elF2α through phosphorylation. The phosphorylated elF2α further activates the activating transcription factor 4 (ATF4) the protein of which translocates into the nucleus transcribing UPR responsive genes necessary to cope up with the cellular stress. The phosphorylated elF2α also arrests the translation of cellular proteins to maintain homeostasis. It has been noted that the AAV particles also activates IRE1 which induces the unusual splicing of X-box binding protein 1 (XBP1) mRNA downstream. The XBP1 protein translocates into the nucleus activating a set of UPR responsive elements. The IRE1 also activates the IKK leading to NF-κ B upregulation. The activated NF-κ B further activates the inflammatory genes thus inducing an inflammatory response (Jayandharan et al., 2011; Balakrishnan et al., 2013). **(D)** Murine leukemia virus (MLV) and UPR. MLV based γ-retroviral vectors, which are the most common used in gene therapy, has been showed to induce neuropathogenecity in astrocytes (Dimcheff et al., 2003). Later in NIH3T3 cells it was shown that the murine retroviruses induce the ER stress related genes such as CHOP/GADD153 which leads to apoptosis (Dimcheff et al., 2003). On the other hand, the lentiviral proteins such as Tat and Nef have been shown to activate unfolded protein response elements (UPRE) by increasing ROS (Tiede et al., 2011; Abbas et al., 2012).

During replication of HSV, there is a rapid generation of large amount of viral proteins that may induce UPR and consequently necessitate modulation of the cellular stress response (Figure 2A). Indeed, a number of HSV-1 proteins have been shown to block phosphorylation of eIF2α, an important stress response mechanism of the cell, which leads to the attenuation of protein synthesis (He et al., 1996; Cassady et al., 1998; Mulvey et al., 2003, 2006, 2007). Cassady et al. (1998) and Mulvey et al. (2003) showed that a HSV viral protein, US11 can repress two kinases (eIF2α, PKR) and PERK upon HSV infection (Figure 2A) (Cassady et al., 1998; Mulvey et al., 2003). He et al., demonstrated that a late protein γ_1_34.5 can dephosphorylate eIF2α with the help of the cellular phosphatase PP1α (He et al., 1996). This inhibition resulted in a 1000-fold increase in the replication efficiency of HSV1 (Talloczy et al., 2006). It has been shown that HSV-1 infection does not activate PERK as well as IRE and was also highly resistant to acute ER stress (Mulvey et al., 2007). This resistance of PERK toward activation by ER stress in HSV-1 infected cells is attributed to the glycoprotein B (gB) associated with the luminal region of PERK (Figure 2A). This study also showed a genetic association between PERK and gB which could regulate the viral protein load in infected cells (Mulvey et al., 2007). To further understand how HSV1 modulates cellular UPR, Burnett et al., reported that HSV-1 can deactivate UPR in the early stages of infection (Burnett et al., 2012). The study observed early repression (less that 24 h post infection) of ATF4 and CHOP due to inhibition of phosphorylation of eIF2α. ICP0, an immediate-early Ad gene product known to have transcription factor capabilities (Yao and Schaffer, 1994), was found to be the primary factor triggering activation of the UPR enhancers during HSV-1 replication, thus helping the virus to sense it at an early stage. Consistent to a previous finding (Mulvey et al., 2003), XBP1(for the IRE1 signaling pathway) remained inactive in this study as well.

### Ad and UPR

Ads are non-enveloped DNA viruses whose genome is comprised of a linear 36 kb double-stranded DNA. The recombinant Ad vectors were first used as a gene transfer agent in 1985 (Ballay et al., 1985; Yamada et al., 1985) and since then have been used as a vehicle for various monogenic diseases (Porteus et al., 2006). For example, Ad vectors expressing cystic fibrosis transmembrane conductance regulator CFTR have been used in phase I clinical studies to treat cystic fibrosis (Zuckerman et al., 1999). Muscular dystrophy is another disease where attenuated Ad vectors have been used to deliver dystrophin cDNA into muscle tissue (Clemens et al., 1996; Haecker et al., 1996; Floyd et al., 1998). Improved Ad vectors have also been used to deliver human coagulation factors VIII and IX for phenotypic correction of hemophilia in preclinical animal models (Zhang, 1999). These vectors have been used in the treatment of several other metabolic and genetic diseases like lysosomal storage disease, phenylketonuria and glycogen storage disease (Amalfitano et al., 1999; Nagasaki et al., 1999; Ziegler et al., 1999; Eto and Ohashi, 2000; Stein et al., 2000; Zingone et al., 2000), neurological disorders like Parkinson/Alzheimer's as well as cardiovascular diseases like atherosclerosis, cerebral ischemia and in cancer therapy (Donahue et al., 2000; Papadopoulos et al., 2000; Choi and Yun, 2013).

As is the case of any foreign protein, Ad vectors are also subject to severe inflammatory response, which lead to their clearance and achieves only transient gene expression in the target tissue. One of the major transcription factor that serves as the mediator of inflammatory response is NF-κ B, which can be activated by various pathological stimuli like bacterial/viral infection and the inflammatory cytokines. It has been shown previously that accumulation of protein load in the ER can activate NF-κ B *via* the PERK and IRE-1 pathway (Tam et al., 2012). It has been demonstrated that the Ad E3/19 K protein can activate NF-κ B mediated by Ca^2+^ release from the ER following a protein overload, *in vitro*. As a result, NF-κ B activates inflammatory cytokines and interferons that constitute the initial anti-viral response of the cells (Pahl and Baeuerle, 1995). As with most viruses, in the late phase of productive infection, viral protein synthesis is promoted in Ad-infected cells while simultaneously inhibiting the cellular protein synthesis. One of the ways Ad does this, is by inhibition of PKR-mediated eIF2α phosphorylation along with the activation of a protein kinase called double-stranded RNA-activated inhibitor (DAI) (Huang and Schneider, 1990). It has also been shown that the non-coding Ad associated RNA molecule I (VAI RNA) can suppress PKR activation by directly binding to it (Mathews and Shenk, 1991; McKenna et al., 2006). VAI RNA is highly expressed during the late phase of Ad infection when it is transcribed by a RNA polymerase III (Soderlund et al., 1976; Thimmappaya et al., 1982; Svensson and Akusjarvi, 1984). It plays a crucial role in preventing shutdown of cellular translational apparatus by inhibiting eIF2α phosphorylation and the activation of PKR, although the precise mechanism remains unclear (Huang and Schneider, 1990). Spergeon et al., have also shown the role of Ad E1B 55-Kd and E4 Open Reading Frame 6 Proteins in promoting its infection in the late phase. These Ad proteins were shown to limit phosphorylation of eIF2α phosphorylation and PKR activation (Figure 2B). This process requires the functioning of the Cul5-mediated E3 ubiquitin-protein ligase of the E1B-55K/E4orf6 complex which is independent of the cytoplasmic levels of VAI RNA (Spurgeon and Ornelles, 2009).

### AAV and UPR

Naturally occurring AAV is small (~22 nm), non-enveloped and contains single-stranded DNA (~4.7 kb). It belongs to the family *Parvoviridae*, and the genus *Dependovirus* (Balakrishnan and Jayandharan, 2014). The genome contains two open reading frames encoding the genes, *rep* (responsible for replication) and *cap* (encodes capsid specific proteins) flanked by a 145 base pair long inverted terminal repeat (ITR) sequence. AAV enters the infective cycle only in presence of other helper virus such as Ad or HSV (Daya and Berns, 2008). Recombinant AAV is produced by stuffing the transgene of interest between the flanking ITRs while the *rep* and *cap* genes are supplied *in trans* along with helper function genes (Wright, 2009). Currently, AAV is the choice vector for many inherited and non-inherited diseases because of its non-pathogenic nature. Another major advantage of using AAV as a gene therapy vector is its low immune profile when compared to Ad vectors (Asokan et al., 2012). So far 12 AAV serotypes (AAV1-12) has been used as gene therapy vectors although several other serovars are known to exist. AAV is naturally hepatotrophic that makes them an attractive choice for liver targeted gene therapy for the treatment of diseases such as hemophilia and alpha1 antitrypsin deficiency (Flotte et al., 2011; Nathwani et al., 2011). However several other alternate serotypes like AAV1, AAV5, AAV9, and rh10 have shown significant promise in targeting tissues like the muscle and the central nervous system (Zincarelli et al., 2008; Tang et al., 2010; Rafi et al., 2012; Gray et al., 2013). Unfortunately, the vector dose-dependent immune response and the presence of pre-existing neutralizing antibody against AAV capsids can limit persistent gene expression in humans (Manno et al., 2006; Boutin et al., 2010). It is known that AAV, after receptor mediated endcocytosis, undergoes trafficking through the endocytic compartments followed by retrograde transport to the Golgi or the ER (Ding et al., 2005). It has been shown earlier that intracellular trafficking of AAV is negatively regulated by components of the ER stress response (Duan et al., 1999; Douar et al., 2001; Ding et al., 2003). For example AAV mediated human factor (F). VIII gene expression improved by ~300–600% upon inhibiting the proteasomal machinery by using pharmacological agents like bortezomib in preclinical animal models of haemophilia (Monahan et al., 2010). Thus, it is quite logical to note that during intracellular trafficking, ER stress could play an inhibitory role in AAV life cycle. Indeed, we have recently shown the role of UPR in AAV infection (Balakrishnan et al., 2013). In this study self-complementary (sc) AAV2 was shown to activate the PERK and IRE-1 pathway in HeLa cells with peak activation 12 h post-infection. ATF6 however was not induced by scAAV2. Interestingly, single-stranded (ss) AAV2 did not induce UPR effectors as dominantly as scAAV2 although it modestly activated PERK and IRE-1. The activation of PERK and IRE-1 was further confirmed by an increased expression of downstream signaling molecules like CHOP and spliced XBP-1, respectively. Inhibiting PERK and or IRE-1 expression in *in vitro* (using shRNA against PERK/IRE-1) and *in vivo* (metformin, i.p) led to a modest increase in gene expression from scAAV2 vectors (Figure 2C). Interestingly, this study also found that alternate AAV serotype vectors like AAV1 and AAV6 can activate distinct arms of UPR. For example, scAAV6 had a comparable effect on PERK activation but not on IRE-1 as scAAV2 vectors. Another observation was the ablation of innate immune response markers following UPR inhibition *in vivo*. This clearly points to the link between UPR activation and clearing of the vectors through innate immune response. It has been shown previously that AAV can activate the classical NF-κ B pathway during the acute phase of infection and trigger downstream inflammatory markers like TNF-α, IL1a, IL6, and leading to the activation of the adaptive immune response (Jayandharan et al., 2011). It is also known that UPR caused by protein overload can activate cellular NF-κ B in the early phase while it is inhibitory in the late phase (Kitamura, 2011) (Figure 2C). Thus, the UPR pathway becomes an important target to reduce inflammatory response in the early stages of AAV infection and to further enhance the persistence and gene expression from AAV vectors. Interestingly, the efficiency of AAV transduction is also known to improve under general cellular stress as shown earlier in cellular models of cystic fibrosis (Johnson et al., 2011).

### Retro-/lenti-virus and UPR

Historically, vectors based on retrovirus which were the first viral vector system described in the early 1980s (Douar et al., 2001) have been the most preferred in clinical gene therapy due to their properties of efficient host DNA integration and persistent gene expression. However, in the clinical trial involving infants with X-SCID, 4 out of 9 patients developed leukemia due to random retroviral integration, this remains a major concern with retrovirus based gene therapy (Cavazzana-Calvo et al., 2000; Kohn et al., 2003). Lentivirus, that belongs to the retroviridae family is also known to facilitate stable integration of the viral genome into the host chromosome. Over the past decade, more than 30 patients with different immunodeficiency disorders have been treated successfully using murine leukemia virus (MLV)-based γ-retroviral vectors to transfer therapeutic genes to autologous hematopoietic cells (Aiuti et al., 2002; Aiuti and Roncarolo, 2009). However, random integration of the lentiviral vectors is also known (Wang et al., 2009a). Although not many studies have been conducted to understand if and how retro- or lenti-viruses combat UPR, there is some evidence that retroviruses can induce ER stress. In Shikova et al. (1993) first showed in cultured astrocytes that neuropathogenicity of MLV viruses may be related to protein misfolding in the ER (Shikova et al., 1993). In Dimcheff et al. (2003) demonstrated that a mouse retrovirus FrCas^E^ is able to induce ER stress related genes like CHOP/GADD153 and Bip *in vitro* in NIH3T3 cells as well as *in vivo* which correlated with the induction of spongiform neurodegeneration (Figure 2D) (Dimcheff et al., 2003). Similarly, mink cell focus-forming murine leukemia virus (MCF13 MLV) has been shown to trigger UPR in mink cells following large accumulation of the viral protein MLV gPr80^env^ *via* upregulation of CHOP proteins (Nanua and Yoshimura, 2004). A Lentivirus-HIV-1 protein called the trans-activator of transcription (Tat) has been reported to induce UPR by increasing reactive oxygen species (ROS) in primary rat striatal neurons indicating that ER stress response could be a critical parameter to control during HIV infection (Figure 2D) (Tiede et al., 2011). Another HIV viral protein called Nef, known to increase infectivity and replication in lymphocytes and macrophages has been shown to directly interact with the eukaryotic elongation factor (eEF)-1α resulting in its cytoplasmic relocalization and the inhibition of stress-induced apoptosis. Conversely, the nuclear re-localization of the Nef/eEF1α complex can decrease mitochondrial cytochrome c release, thereby inhibiting the caspase activation. This mechanism demonstrates how the lentivirus (HIV) can prevent cell death under conditions of stress condition yet is able to create an environment favoring optimal viral replication (Abbas et al., 2012) (Figure 2D). Another unique retrovirus called Foamy viruses (FVs) have also been extensively studied as a gene therapy vector due to their lack of pathogenicity, broad tissue tropism and the ability to carry large (minimum ~9.2 kb) transgenes (Heneine et al., 2003; Trobridge, 2009). Hematopoietic stem cell (HSC) gene therapy is one area where FVs have been extensively evaluated with considerable success (Josephson et al., 2004; Bauer et al., 2008). However there are no published evidence which have studied the interaction between FVs and cellular UPR. However it is possible that like other retro-/lenti- viruses, FVs would have developed mechanism to either counteract or utilize the UPR machinery to enhance its own replication in the infected cells.

### Other viral vectors and UPR

In addition to the commonly used viral vectors described above, attempts have been made to utilize other viruses as vectors for certain disease conditions. For example, *Vaccinia viral vectors* have been in use as a potential therapeutic for cancer gene therapy (Yu et al., 2009; Seubert et al., 2011) mainly because of its efficient infection and gene expression in a wide range of difficult to transduce tumors (Yu et al., 2004) as well as their inability to integrate into the chromosome (Shen and Nemunaitis, 2005). Also, the safety profile of Vaccinia virus as a therapeutic agent is well understood due to its long and widespread use as a vaccine for small pox in humans. Like most viruses, vaccinia virus also regulates the cellular UPR machinery to facilitate its infection. For example, a vaccinia viral protein K3L which has ~28% sequence identity with eIF-2a is thought to function as a pseudo substrate for its kinase, thus blocking the PKR activity and leading to the inhibition of PERK and eIF2α molecules (Sood et al., 2000). Following ER stress response, another Vaccinia protein called F1L can indirectly inhibit the activation of the apoptotic protein *Baxby* by interacting with the proapoptotic BH3-only proteins through Bak and Bax (Taylor et al., 2006).

*Varicella zoster virus (VZV)*, the causative agent of varicella (chickenpox) and zoster (shingles) and a member of the *Herpesviridae* family has also been tested as a cancer gene therapy vector (Degreve et al., 1997). VZV has been shown to induce cellular UPR through ER stress *in vitro*. It has been shown to activate both the IRE-1 and the CHOP pathway and ultimately leads to autophagy (Carpenter et al., 2011). This study also confirmed that the VZV structural glycoproteins—gE (ORF68), gI (ORF67), gH (ORF37), and gL (ORF60) were enough to induce UPR during an active viral infection.

*Epstein–Barr virus (EBV)* is a member of the *Herpesviridae* family that has a natural tropism for B cells. This property of the virus has been utilized to deliver GM-CSF to human B cells from B-cell chronic lymphocytic leukemia (B-CLL) patients as a potential immune therapy (Hellebrand et al., 2006). However, since EBV is associated with a number of human malignancies, rigorous vector modification and validation is called for prior to its application as a gene delivery vehicle in humans. The latent membrane protein 1 (LMP1) oncogene of EBV is shown to induce the phosphorylation of eIF2α by activating all the three arms of UPR, the PERK, IRE-1, and ATF6 pathways. This activation in turn up-regulates LMP1, which leads to induction and maintenance of the proliferating B lymphocytes (Lee and Sugden, 2008). Thus it seems that the UPR pathway is required for EBV to enter into its lytic stage toward maintaining its proliferative and infectious life-cycle (Taylor et al., 2011).

*Sendai virus (SeV)* is a negative strand RNA virus which utilizes sialic acid residue or a sialoglycoprotein as their receptor for cell entry (Markwell et al., 1981). The major advantages of using recombinant SeV as a gene therapy vector is its non-pathogenicity and the ability to be generated in high titre during packaging process. Preclinical studies have shown that SeV can transduce different cell types like vascular tissue (Masaki et al., 2001), skeletal muscle (Shiotani et al., 2001), airway epithelial cells (Yonemitsu et al., 2000) and synovial cells (Yamashita et al., 2002), quite efficiently. Further, it has been shown that SeV vector can efficiently transfer its cargo to CD34^+^ cell and CD34^+^ cell subpopulations derived from human cord blood (Jin et al., 2003). In addition, SeV was also able to get stable gene expression in myeloid, erythroid or mixed progenitor cells (Jin et al., 2003). However, since SeV induces cytopathic effects in infected cells, toxicity concerns remain. SeV has been shown to upregulate CXCL2 protein following ER stress, which can lead to cell death via activation of caspase-8 and caspase-3 mediated apoptosis (Bitzer et al., 1999; Versteeg et al., 2007). It is also thought that this virus can activate eIF2α kinases like PERK and PKR to induce IFN regulatory factor (IRF) 7, a major player in host antiviral innate response. ATF4, another key regulator of cellular response to viral infection can be upregulated *via* phosphorylation of eIF2α and the activation of IRF7 ultimately helping in cellular recovery (Liang et al., 2011).

*Alphavirus vectors based on Sindbisvirus (SINV) and Semliki Forest virus (SFV) is* another group of virus that has been evaluated as a gene therapy vector because of advantages like broad host range, efficient replication in the cytoplasm and the capacity to produce high levels of recombinant proteins. Several preclinical studies have been conducted so far to evaluate the efficiency of alphaviruses as a gene transfer vehicle. For example, SFV was shown to transduce cardiovascular cells as well as human tumor cells to deliver IL-12 (Roks et al., 1997; Zhang et al., 1997). Alphaviral vectors have also been looked upon as a potential delivery vehicle for DNA-based vaccines (Berglund et al., 1997, 1998). More recently, SFV was used for gene transfer into the central nervous system but was also toxic (Graham et al., 2006). In mammalian cells, SFV envelope glycoproteins activate the UPR response through induction of CHOP proteins and its consequent upregulation of caspase-3, caspase-8, and caspase-9 apoptotic enzymes (Barry et al., 2010). SFV has also been shown to have delayed RNA synthesis in the presence of Brefeldin A, a potent UPR inducer (Molina et al., 2007). Another report suggests that PERK can suppress SFV viral replication at an early stage by eliciting strong interferon response in the mouse brain (Barry et al., 2009). One study revealed that the alpha virus SINV could activate PERK and IRE-1 but not the ATF6 within 48 h of infection *in vitro*. In this study, SINV uncontrollably activated the UPR by phosphorylation of eIF2α and leading to apoptosis (Rathore et al., 2013). Moreover SINV has been shown to promote autophagy in neuronal cells both in *in vitro* and *in vivo* probably via activation of the UPR pathways, thus limiting the spread of viral infection (Orvedahl et al., 2010; Shi and Luo, 2012).

## Strategies to inhibit UPR against viruses used in gene transfer

One of the ways to inhibit UPR is through small molecule inhibitors, which can repress the cellular proteasomal machinery. For example, the use of pharmacological agents that inhibit proteasomes like metformin, MG-132, and ricin has been previously shown to reduce cellular UPR (Lee et al., 2003; Parikh et al., 2008; Amanso et al., 2011; Theriault et al., 2011). We have previously shown that scAAV2 upregulates PERK and IRE-1α genes in murine liver, ~24 h post vector administration (Balakrishnan et al., 2013). This effect was reversed when animals were pretreated with metformin (250 mg/kg). More importantly, it was also found that attenuation of the UPR response against AAV also inhibited the expression of various inflammatory cytokines and chemokines like Chemokine (C-C motif) ligand 12 (Ccl12), Chemokine (C-C motif) ligand 11 (Ccl11), Chemokine (C-C motif) ligand 22 (Ccl22), Chemokine C-X-C motif ligand 13 (CXCL13), Chemokine (C-C motif) ligand 24 (Ccl24), Chemokine C-C motif receptor 2 (Ccr2) and chemokine C-X-C motif ligand 15 (CXCL15) (Balakrishnan et al., 2013). Thus, inhibiting the UPR by proteasomal repressors could potentially reduce innate immune response against AAV leading to higher and probably persistent gene expression. However, it is a known fact that systemic administration of proteasomal inhibitors can have adverse effects (Rajkumar et al., 2005). It is however conceivable that transient inhibition of UPR pathways prior to gene transfer and during the initial period of viral infection might lead to improved gene transfer efficiency. Another way to repress UPR could be by the use of silencing (si) RNAs against specific components of the UPR pathway. Our study had previously shown a modest increase in transgene expression from AAV vectors when the PERK and IRE-1α pathways were inhibited by specific siRNA *in vitro* (Balakrishnan et al., 2013). shRNAs against UPR components can also be potentially tested *in vivo*. Such shRNAs can be delivered under inducible promoters to avoid adverse effects caused by long term suppression of UPR machinery.

To avoid immune mediated clearance of viral vectors during gene therapy, ideally the vector dose should be kept to the minimum. This would allow the vectors to not only to escape the host immune surveillance before entering the target cells, but may also reduce cellular stress. To this end, vector bioengineering becomes a very important tool by which novel, optimized vectors can be created as described earlier with AAV (Markusic et al., 2010; Qiao et al., 2010; Gabriel et al., 2013; Sen et al., 2013a,b) to achieve efficient gene transfer at lower vector doses.

## Conclusions

In higher eukaryotes, UPR is a beneficial process that protects the cell from undue stress. Cellular UPR strives to reduce the burden on the ER by enhancing its capacity with the help of several stress response chaperones. If this process becomes futile, UPR can induce apoptosis of the host cell. Most viruses reprogram the cellular translational machinery to facilitate the generation of their proteins, but this process can also trigger the UPR pathways, which consequently may lead to cell death. For successful gene therapy, the survival of the transduced cells is very important to achieve sustained gene expression. In this scenario, transient inhibition of UPR prior to gene transfer, by strategies discussed above, provides an attractive alternative to improving the safety and efficiency of viral gene therapy. However, further detailed understanding of the sub-cellular processes that activate UPR against such viral vectors is also necessary to tailor specific strategies and to shift the balance in favor of virus persistence in the host without compromising either of their survival.

### Conflict of interest statement

The authors declare that the research was conducted in the absence of any commercial or financial relationships that could be construed as a potential conflict of interest.
